# Reelin Signaling in Neurodevelopmental Disorders and Neurodegenerative Diseases

**DOI:** 10.3390/brainsci13101479

**Published:** 2023-10-19

**Authors:** Aurelie Joly-Amado, Neel Kulkarni, Kevin R. Nash

**Affiliations:** Department of Molecular Pharmacology and Physiology, Morsani College of Medicine, University of South Florida, 12901 Bruce B Downs Blvd, Tampa, FL 33612, USA; neelkulkarni@usf.edu (N.K.); nash@usf.edu (K.R.N.)

**Keywords:** Reelin, aging, neurodegenerative diseases, neurological disorders

## Abstract

Reelin is an extracellular matrix glycoprotein involved in neuronal migration during embryonic brain development and synaptic plasticity in the adult brain. The role of Reelin in the developing central nervous system has been extensively characterized. Indeed, a loss of Reelin or a disruption in its signaling cascade leads to neurodevelopmental defects and is associated with ataxia, intellectual disability, autism, and several psychiatric disorders. In the adult brain, Reelin is critically involved in neurogenesis and synaptic plasticity. Reelin’s signaling potentiates glutamatergic and GABAergic neurotransmission, induces synaptic maturation, and increases AMPA and NMDA receptor subunits’ expression and activity. As a result, there is a growing literature reporting that a loss of function and/or reduction of Reelin is implicated in numerous neurodegenerative diseases. The present review summarizes the current state of the literature regarding the implication of Reelin and Reelin-mediated signaling during aging and neurodegenerative disorders, highlighting Reelin as a possible target in the prevention or treatment of progressive neurodegeneration.

## 1. Introduction

### 1.1. Reelin Protein

Seventy-five years ago, a mouse was born in Edinburgh with a unique behavioral phenotype. The mouse walked with a swaying motion that caused it to collapse to its side, alternating from one side to the other. Given that this behavior was “remarkably suggestive of inebriation” it was designated as the *reeler* mutant and breeding revealed a recessive mutation [1]. Subsequent analysis of *reeler* brains identified severe distortions of the cytoarchitectonic lamination characteristic of regions such as cerebral cortex, hippocampus, and cerebellum due to disordered neuronal migration during development [2]. Thirty years later the disrupted gene that caused the *reeler* phenotype was identified and designated as Reelin (RELN; [3]).

**Figure 1:**
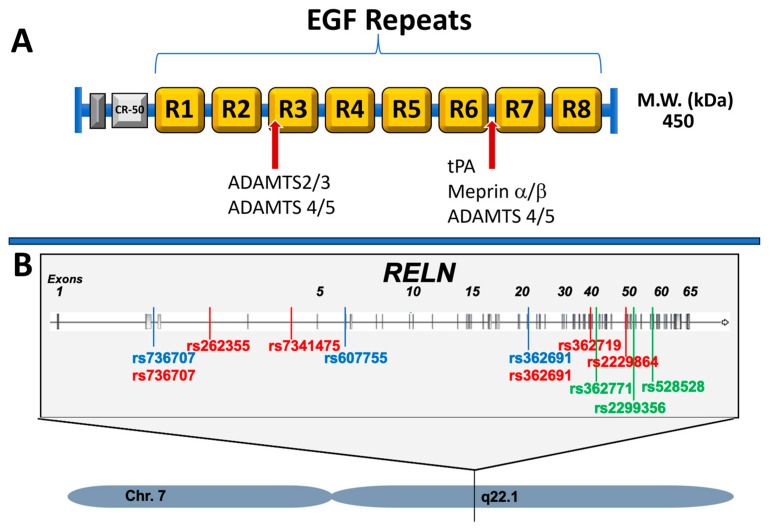
(**A**) Schematic representation of the Reelin protein and the proteases involved at its two main sites of cleavage: these include the plasminogen activator (tPA), the Meprin α/β, and the A disintegrin and metalloproteinase with thrombin motifs (ADAMTS). (**B**). Schematic representation of single nucleotide polymorphisms (SNPs) of the human RELN gene involved in schizophrenia (red), autism (blue), and Alzheimer’s disease (green). References are in the text in the respective sections. Exact locations are as follows: Variation:rs262355: Location:Chr7: 103,785,668; Variation:rs7341475: Location:Chr7: 103,764,368; Variation:rs607755: Location:Chr7: 103,749,507; Variation:rs362691: Location:Chr7: 103,610,714; Variation:rs362771: Location:Chr7: 103,548,441; Variation:rs362719: Location:Chr7: 103,545,430; Variation:rs2229864: Location:Chr7: 103,515,258; Variation:rs2299356: Location:Chr7: 103,669,375; Variation:rs528528: Location:Chr7: 103,748,638; and Variation:rs736707: Location:Chr7: 103,489,956. Source variation viewer https://www.ncbi.nlm.nih.gov/variation/view (accessed on 11 October 2023).

Reelin is a large extracellular matrix protein which consists of eight epidermal growth factor (EGF)-like domains [3,4] that are proteolytically processed in vivo at two main sites of cleavage, within repeat 3 and between repeats 6 and 7 [5,6], leading to the N-terminal, central, and C-terminal fragments (Figure 1A). Many proteases have been identified as being able to cleave Reelin: plasminogen activator (tPA), Meprin α/β, and A disintegrin and metalloproteinase with thrombin motifs (ADAMTS) 4 and 5 have been identified as proteases of the C-terminal cleavage site [7,8,9]. ADAMTS 2 and 3 have been shown to cleave at the N-terminal cleavage site [10,11]. It should be noted that most of these proteases have not been fully validated with in vivo studies, apart from ADAMTS 3, which showed a loss of N-terminal cleavage in an ADAMTS 3 knockout mouse [11]. Additionally, Quattrocchi et al. proposed that Reelin itself may have protease activity and induce self-cleavage [12], but others have failed to replicate these results [13]; hence, further investigation is needed. Reelin processing was recently reviewed by Hattori and Kohno (2021) [14]. The current understanding would suggest that, perhaps, cleavage is regulated in a temporal or brain region in a specific manner with contribution from different proteases at different stages of development or in different brain areas. Immunohistochemical labeling using the monoclonal G10 antibody demonstrates that Reelin-expressing neurons are located in the hilar region of the dentate gyrus, stratum oriens, stratum radiatum, and the stratum lacunosum-moleculare layers of the hippocampus of the adult brain [15,16] (reviewed in [17]). The Reelin receptors very-low-density lipoprotein receptor (VLDLR) and low-density lipoprotein receptor-related protein 8 (LRP8), also known as apolipoprotein E receptor 2 (ApoER2), are both found in the cerebellum and the hippocampus. In the cerebellum, VLDLR is found in the Purkinje cell layer, and ApoER2 is expressed in the internal granule layer. In the hippocampus, VLDLR was identified in the stratum radiatum, and ApoER2 was found in the hippocampal multipolar cell accumulation zone [18].

Reelin was originally studied because of its importance in brain development during embryogenesis [19,20]. During development, Reelin is expressed by the Cajal–Retzius cells located in the hippocampus and cortex and by the granule cells located in the cerebellum [18,21,22]. Post-development, Reelin expression slows down in the Cajal–Retzius cells and becomes more prominent in the GABAergic interneurons [23]. Early knowledge of Reelin signaling comes from the developmental examination of the central nervous system (CNS) when Reelin, Reelin receptors, or the major Reelin intracellular protein, Disabled-1 (Dab1), are absent [24,25,26,27]. *Reeler* mice display a roughly inverted cortical lamination pattern, with an excess of cells in the normally cell-sparse layer I or marginal zone [28]. The phenotype of mice that lack both receptors is virtually indistinguishable from that of *reeler* animals [26]. Mice with mutations of the gene *disabled-1*, also known as *scrambler* mice (*Dab1scm*), are characterized by a loss of function of the disabled-1 adaptor protein, with a reduced expression of Dab-1 to 5% of the wild-type level [29]. Although Reelin expression is normal in these mice, the brain histopathology of *Dab1scm* appears identical to that of *reeler* mice. *Dab1scm* mutants also exhibit ataxia, tremors, and imbalance, as well as impaired spatial learning in the Morris water maze and severe deficits in motor coordination [30,31]. These studies highlight the necessity of Reelin, Reelin receptors, and the intracellular adapter protein for proper neuronal migration in the developing brain. After introducing Reelin and Reelin signaling, this review will focus on the role of Reelin in the adult brain and in neurodegenerative disorders. The peripheral effects of Reelin, although of significant importance, will not be discussed here (for review see [32]).

### 1.2. Reelin Signaling

**Figure 2:**
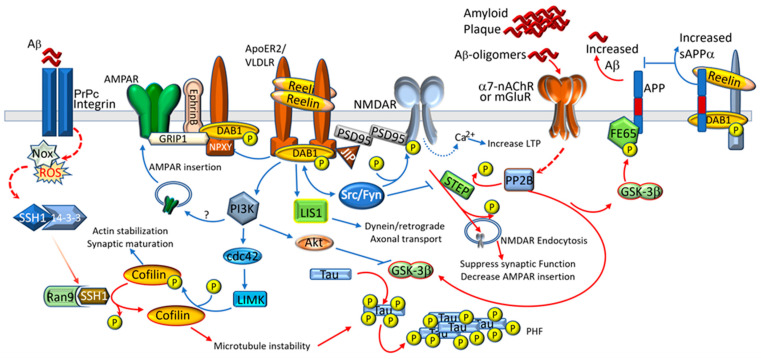
Dysregulation of Reelin signaling in Alzheimer’s disease. Reelin signaling acts in opposition to the effects of amyloid, reducing tau phosphorylation and improving synapse stability. Abbreviations: Aβ (Amyloid Beta), APP (Amyloid precursor protein), ApoER2 (ApoE receptor 2), Dab1 (Disabled-1), FE65 (APP-binding family B member 1-APBB1), GRIP1 (Glutamate receptor-interacting protein 1), GSK-3β (Glycogen synthase kinase 3 beta), JIP (JNK-interacting protein), PI3K (phosphatidylinositol-3-kinase), PP2B (protein phosphatase-2B), PrPC (cellular prion protein), PSD-95 (Postsynaptic density protein 95), LIMK (LIM Kinase), LIS1 (platelet-activating factor acetylhydrolase IB subunit alpha), Ran9 (Ran binding protein 9), SSH1 (Slingshot Protein Phosphatase 1), STEP (striatal-enriched protein tyrosine phosphatase), and VLDLR (very-low-density lipoprotein receptor).

The discovery of the signaling capabilities of the Reelin receptors during developmental laminar formation expanded insight into Reelin signaling in the postnatal brain. Reelin binds to two lipoprotein receptors, VLDLR and ApoER2 (or LRP8), causing the receptors to cluster, or at least dimerize [24,33,34]. Receptor clustering leads to the recruitment and tyrosine phosphorylation of Dab1, via the Src family kinases (SFKs) [33,35,36]. The phosphorylation of Dab1 leads to the activation of multiple downstream signaling pathways. These pathways include the activation of the Src family of tyrosine kinases [37,38,39], protein kinase B (AKT) [38], phosphatidylinositol-3-kinase (PI3K) [20,40], extracellular signal-regulated kinase (Erk) [41] pathways and the cyclin-dependent kinase pathway [42,43,44,45] (Figure 2). Tyrosine-phosphorylated Dab1 also interacts with the microtubule associated protein, lissencephaly type1 (LIS1) [46], which is critical for neuronal migration [47]. LIS1 is also an important player in retrograde mitochondrial transport through its interactions with the microtubule cytoskeleton [48].

As addressed above, Reelin can be proteolytically cleaved at two sites within the protein. It has been proposed that this cleavage may regulate the function of Reelin. For example, cleavage has been reported to reduce Reelin multimerization and reduce Reelin’s ability to induce Dab1 phosphorylation and downstream signaling [11,14,49,50,51,52]. Studies have suggested that the Reelin’s C-terminal region is important for Dab1 phosphorylation, dendrite development, and the positioning of cortical neurons [53,54,55]. However, studies using the central Reelin fragment (R3-6 region) have also shown that this fragment can elicit downstream activation by binding to VLDLR and ApoER2 similarly to full-length Reelin [5,56]. Further work is required to clarify these conflicting findings on the activity and importance of Reelin’s proteolytic cleavage. It may be that differences in the experimental design, such as the cells used in these different studies, or in the preparations of the Reelin protein used may affect the outcomes of these studies.

Reelin activation can lead to effects on cell adhesion, dendrite outgrowth, and spine formation, as well as synaptic receptor reorganization. Reelin binding to VLDLR and/or ApoER2 leads to modulation of synaptic function and cognition by activating numerous neuronal signal transduction pathways in the adult brain [27,57]. The activation of Src and Fyn leads to tyrosine phosphorylation of the NR2 subunits of N-methyl-D-aspartate receptors (NMDARs), which reduces NMDAR endocytosis and also results in increased Ca^2+^ influx when those receptors are activated [27,58]. De novo insertion of the α-amino-3-hydroxy-5-methyl-4-isoxazolepropionic acid receptor (AMPARs) at the synapse is dependent on the adaptor glutamate-receptor-interacting protein 1 (GRIP1) and ApoER2 signaling. GRIP1 binds ApoER2 in a complex with ephrinB2 and the AMPA glutamate receptor 2 (GluR2) subunit of the AMPARs upon Reelin stimulation and induction of neuronal activity, leading to AMPAR membrane insertion and long-term potentiation (LTP) [59]. The modulatory actions of Reelin in the hippocampus has established Reelin as an important player in hippocampal function.

In the hippocampus, Reelin and its receptors are required for the proper function of synaptic transmission and plasticity. Reelin supplementation or overexpression potentiates glutamatergic neurotransmission, LTP, and synaptic maturation and increases AMPA and NMDA receptor expression, synapse membrane localization, and activity [57,58,60,61,62,63]. Reelin favors the substitution of NR2B by NR2A subunits at the synapses, which enhances LTP [60], and chronic treatment with Reelin reduces the number of silent synapses in vitro [64]. Furthermore, Reelin has been shown to increase GABAergic neurotransmission, with increases in glutamic acid decarboxylase (GAD) [57,65]. In addition, a 50% reduction of Reelin in heterozygous *reeler* mice (HRM) results in the disruption in learning and memory, dendritic spine maturation, and LTP [65,66,67].

Consistently, hippocampal synaptic plasticity is significantly increased following in vivo supplementation via a bilateral Reelin injection in genetically unaltered wild-type mice. The examination of dendritic spines after a single Reelin injection shows increased spine density similar to that seen in organotypic and acute hippocampal slices [57]. Furthermore, an increase in both associative and spatial learning and memory was observed 5 days after injection. Recombinant Reelin’s measurable output in improving CNS function was exemplified in other animal models of disease (discussed below).

## 2. Reelin in Aging and Disease

Reductions in Reelin levels and signaling have been found during aging, as well as having been associated with a number of neurological diseases, including ataxias, Alzheimer’s disease (AD), schizophrenia (SZ), and also being partially involved in autism and even traumatic brain injuries (TBI) [68,69,70,71]. In humans, a loss of Reelin results in a type of lissencephaly with severe cortical and cerebellar malformation [72]. Reelin and its association with aging and different neurological diseases is discussed below.

### 2.1. Aging

In addition to its importance during embryogenesis, it is now well established that Reelin plays a critical role in adult synaptic function and plasticity and is essential for normal brain function throughout life (for review [16]). Therefore, it should not come as a surprise that lower levels of Reelin are associated with cognitive impairments during aging. Indeed, recent studies have shown an age-related reduction in Reelin expression in the hippocampus of wild-type mice [73]. Similar observations were made in aged rats and non-human primates exhibiting memory impairments [74,75], suggesting that a loss of Reelin may induce synaptic dysfunction during aging-related memory decline. The disruption in the expression of Reelin or of its receptors results in associative and spatial learning defects, impairment of hippocampal LTP, and immature dendritic spine morphology in animal models [26,27]. Additionally, the interruption of Reelin signaling through the administration of the recombinant receptor-associated protein (RAP) into the entorhinal cortex of a small group of young rats led to impairments in their spatial memory during Morris’ water maze, which were associated with decreased levels of synaptophysin [74].

Although the specific mechanisms of decreased Reelin during aging are yet to be unraveled, studies in rodents and primates show that Reelin accumulates into aggregates and, eventually, into plaques in the hippocampus during normal aging [73]. The combination of this aggregation with a decrease in Reelin-expressing neurons with age would lead to decreased levels of soluble Reelin, leading to age-related memory impairments. Interestingly, Reelin plaques were highly increased in a mouse model of AD and colocalized with amyloid-β (Aβ) plaques [73]. Further studies from the same team established that abnormal oligomeric Reelin deposits in the hippocampus during aging could potentially create a precursor condition for fibrillary Aβ plaque formation [73,76]. Reelin plaques in the aged wild-type mice were particularly found around hippocampal afferent Gamma-aminobutyric acid (GABA)-ergic neurons and were associated with astrocytosis. Astrocyte activation was observed with increased inclusion of small intracellular Reelin-positive granules, while larger deposits seemed to remain extracellular. This suggests a tentative clearance from the glial cells of the small Reelin aggregates, while the uncleared larger deposits might affect the normal function and survival of afferent neurons, leading to neurodegeneration in the hippocampus and subsequent memory impairments [77]. Thus, brain levels of Reelin and, specifically, Reelin aggregates could be a possible biomarker of neurocognitive aging and need to be considered as a possible target in the treatment of neurodegenerative diseases.

### 2.2. Reelin in Ataxias

Ataxia is characterized by the progressive neurodegeneration of the cerebellum or its efferences, which result in the dysregulation of motor coordination. Sporadic ataxia can be caused by many conditions such as head trauma, autoimmune diseases, and infections. Hereditary ataxias can be autosomal-dominant (spinocerebellar ataxias, episodic ataxia) or recessive (Friedreich’s ataxia, ataxia telangiectasia, congenital ataxia, and Wilson’s disease) [78]. The link between Reelin and ataxia comes from the observation of the ataxia phenotype in a mouse model of Reelin deficiency, the *reeler* mouse [3]. Ataxia in the *reeler* mouse is thought to be caused by cell death and neurodegeneration in the cerebellum, especially in granule cells [79,80]. The ectopic expression of Reelin in the cerebellum of *reeler* mice induced the restoration of the tyrosine phosphorylation of Dab1, which was sufficient to rescue purkinje cells’ migration and was associated with the partial rescue of the ataxia phenotype [81]. Similarly, the injection of Reelin protein into the cerebellum of *reeler* mice ameliorated motor function [82]. In addition, decreased levels of Reelin, together with early deficits in Reelin signaling, have been evidenced in the cerebrospinal fluid of patients with Ataxia-telangiectasia (A-T), and a decreased expression of the Reelin receptor was shown in a mouse model of A-T (Atm-/-) [83]. A-T is a rare, inherited debilitating disorder that affects the nervous, immune, and other bodily systems and is characterized by progressive ataxia beginning in early childhood, with difficulty walking, problems with balance and hand coordination, as well as neuropathy. A-T is clinicopathologically defined by the degeneration of cerebellar Purkinje neurons (PNs), which is caused by mutations in the ATM (ataxia telangiectasia mutated) protein [84], a serine-threonine kinase mainly known for its role in the DNA double-strand break response [85,86]. Interestingly, the ATM protein is not expressed in PNs, and how DNA damage relates to the preferential neurodegeneration of PNs is poorly understood. Studies now show that the ATM protein is found in cerebellar granules neurons (GN) that project to PNs. In the A-T cerebellum, early pathology is detected in the GN axons and leads to cell death, which, in turn, affects the PNs through dendritic inputs leading to anomalies in both cell types. Reelin is secreted from the cerebellar granule neuron pre-synapses and plays a role in PNs’ radial migration, excitability, and dendritic differentiation [66]; so, decreased Reelin levels in the A-T are likely to be involved in PNs degeneration. The loss of Reelin would contribute to early loss of dendritic spine maturation and this likely reduced functionality of PNs.

Reelin has also been evidenced as a target in spinocerebellar ataxia. Spinocerebellar ataxia (SCA) is a heterogeneous group of neurodegenerative ataxic disorders with autosomal dominant inheritance with more than 40 genes [87]. Many SCAs are caused by CAG nucleotide repeat expansions that encode polyglutamine. Spinocerebellar ataxia type 7 (SCA7) results from the polyglutamine expansion of the ataxin-7 protein and leads to pathology primarily in the retina and in the cerebellum, with the degeneration of cerebellar PN. A reduction of Reelin gene expression (around 70% decrease) was observed in mutant SCA7 astrocytes, together with a decrease in Reelin protein levels [88]. Reelin signaling dysregulation was also observed in the cerebellum of patients with SCA37 (spinocerebellar ataxia type 37) [89] and was attributed to a mutation in the *Dab1* chromosomal region [90,91]. These reports show that, although Reelin is not directly mutated in ataxias, it does play a significant role in different forms of ataxias and, thus, suggests that Reelin supplementation or enhanced Reelin signaling could be a potential therapeutic target.

### 2.3. Alzheimer’s Disease

Alzheimer’s disease (AD) is a progressive neurodegenerative disease, which is characterized by the formation of extracellular amyloid plaques (Aβ or senile plaques) and intracellular neurofibrillary tangles resulting from tau protein hyperphosphorylation and aggregation. Pathological changes in tau and Aβ have been linked to the endocytosis of AMPARs and NMDARs, leading to synaptic dysfunction, impaired LTP, and cognitive decline [92,93]. Recently, a whole genome analysis identified the Reelin pathway (DAB1-RELN) to be associated with Alzheimer’s disease pathogenesis [94]

Reductions in Reelin appear to be an early feature of AD, with reductions reported for brain regions that are affected in AD subjects and Aβ transgenic mouse models [95,96,97,98,99]. However, late in AD progression there appears to be an increase in Reelin levels [100,101,102,103] and increased RELN mRNA in the brain of patients with AD [101]. This increase in Reelin could be a compensatory mechanism since it is associated with a decrease in signaling [104]. In this regard, AD could be characterized by a Reelin-resistant state, where compensatory, increased levels of Reelin in the brain would not be sufficient to induce Reelin signaling and the subsequent synaptic plasticity and memory processes. Increased Reelin levels could also be associated with decreased free Reelin. Indeed, immunoprecipitation of Aβ from AD brain tissue pulls down Reelin and vice versa, suggestive of a strong interaction that likely leads to the inhibition of normal Reelin function [104]. Also, Reelin-enriched proteins aggregates have been found in the hippocampus in aged rodents and non-human primates [73]. These Reelin aggregates were also observed to colocalize with non-fibrillary amyloid-plaques [73]. Further studies should explore whether an increase in Reelin levels is associated with an increase in Reelin aggregates and a decrease in soluble Reelin.

Reelin levels were shown to be increased in the brains of APP knockout mice [95]. Transgenic mice overexpressing Reelin (TgRln) crossed with mice overexpressing hAPP_Swe/Ind_ (J20) demonstrated delayed fibril formation; but, more importantly, they showed that Reelin overcomes the toxicity of Aβ oligomers, rescues dendritic spine density, and enhances cognitive performance of the J20 mice [105]. Conversely, reduced Reelin signaling has been shown to accelerate Aβ plaque formation and tau pathology [98,106,107]. In contrast to these findings, Lane-Donovan et al. found that, although the loss of Reelin resulted in severe memory impairment, this impairment was not a result of accelerated amyloid plaque deposition [108]. These data support the idea that Reelin and amyloid could have opposing effects on the disease’s pathology phenotype.

In support of Reelin’s potential involvement in AD, three polymorphisms of variants in the RELN gene (loss of function) have been associated with Alzheimer’s disease (Figure 1B) [109,110]. More interestingly, there has been a recent association of a RELN variant with reduced penetrance of the presenilin-1 (PS1) mutation in the Columbian kindred [111]. In one PS1 case, the RELN variant H3447R (COLBOS) was associated with a delayed onset of cognitive impairment of over 20 years. The amyloid pathology in this case was higher than the typical PS1 cases lacking this variant, suggesting a longer period of amyloid accumulation in the incident case. However, the amount of tau pathology was in the low range compared to other cases in the kindred, suggesting some delay or slowing of the tauopathy associated with the RELN variant. The authors conclude that the variant has a gain of function relative to the common RELN variant. Knock-in mouse models demonstrate that the H3447R variant increases Reelin signaling as measured by an increase in the phosphorylation of Dab1; yet, ELISA binding to the receptors was unaffected. When crossed with the JNPL3 tauopathy mouse, the H3447R variant reduced the accumulation of phosphorylated tau and reduced the abnormal tail elevation response. Overall, the data presented suggest that the presumably protective RELN variant increases signaling and slows tauopathy, thereby delaying cognitive impairment in the described cases. Further studies are needed to investigate the gain of function of this new variant and its relation to amyloidosis and tauopathy, as understanding this variant may give insight into how to increase Reelin signaling for therapeutic interventions.

Lack of Reelin and defective Reelin signaling are associated with increased tau phosphorylation [24,112,113]. A decline in the levels of Reelin in the lateral entorhinal cortex of aged rats with cognitive impairment has also been observed. These rats exhibited changes in other molecular markers, including an increased accumulation of phosphorylated tau and a decreased synaptophysin immunoreactivity [74]. In primary cultures, Aβ treatment is associated with impaired Reelin signaling leading to Reelin being less capable of down-regulating tau phosphorylation via Dab1 and Glycogen synthase kinase-3 beta (GSK3β) kinase [113]. This may be due to alterations in Reelin processing and glycosylation [100,103,114] or the inefficiency of Reelin to form active homodimers and, thus, its reduced ability to bind efficiently to its receptor, ApoER2 [113]. Recent studies also showed that the overexpression of Reelin in a mouse model of tauopathy (TgRln/VLW mice) led to a reduction in tau phosphorylation independently of the total tau, together with an improvement in LTP and cognition [115]. Furthermore, the addition of the Reelin protein in the primary hippocampal neurons of mice prevented an Aβ-derived diffusible ligands (ADDLs)-induced migration of tau neurofilaments to the dendrites [115]. The Reelin conditional KO in the tauopathy mouse model PS19 led to the exacerbation of age-related spatial cognitive impairments [116].

Reelin and AD pathologies appear to have opposite effects on synaptic plasticity. In AD, Aβ binds to a number of cellular receptors including α/7 nicotinic acetylcholine receptors and metabotropic glutamate receptors, in particular mGluR5 [117,118,119]. This results in the activation of calcineurin or protein phosphatase-2B (PP2B) which, in turn, regulates the levels and activity of striatal-enriched protein tyrosine phosphatase (STEP) [120]. STEP is increased in AD brains and animal models [121]. The Aβ-mediated activation of STEP leads to an excessive dephosphorylation and internalization of NMDARs [120,122]. STEP activation can also inactivate Fyn kinase, a kinase critical in phosphorylating NMDARs to retain them at the cell membrane [123,124,125]. Notably, increased Reelin signaling can reduce STEP activity through the activation of Src/Fyn, thus preventing the dephosphorylation of the NMDAR subunits Glu2NB and AMPAR GluA2 which leads to increasing membrane retention of the NMDARs [126,127]. In 3xTg-AD mice, the inhibition of STEP recovered cognitive deficits but had no effect on pathology [128]. Thus, Aβ and Reelin can act in opposition (Figure 2), and it is possible that the interaction of Reelin with Aβ is a defense mechanism where the retention of Aβ by Reelin would prevent its association with acetylcholine and glutamate receptors, thus preventing NMDAR endocytosis and the subsequently decreased synaptic function.

The ability of Aβ to inhibit hippocampal LTP provides a cellular correlation for its action on learning and memory. This inhibition of LTP appears to involve a signaling pathway with caspase 3, Akt1, and Glycogen synthase kinase 3 beta (GSK3β) for this effect in rats and mice [129]. GSK3β transient over expression or activation by wortmannin reduces synaptic LTP and provides a potential biochemical mechanism through which Aβ can induce tau hyperphosphorylation [130]. Importantly, the application of Reelin to hippocampal slices prevents the reduction in LTP induced by Aβ [126]. This may in part be due to Reelin’s ability to reduce GSK3β activity but also to its ability to increase synaptic strength. Aβ-induced synaptic dysfunction is dependent on the overstimulation of NMDARs resulting in the elevation of cytoplasmic Ca^2+^, which, in turn, triggers downstream pathways (involving phospho-tau (p-tau), postsynaptic density protein 95 (PSD95), PP2A, Gsk-3β, Fyn, cofilin, and calcium–calmodulin (CaM)-dependent protein kinase II CaMKII) and causes the endocytosis of AMPARs as well as NMDARs [92,131,132,133,134,135,136]. The removal of AMPARs from synaptic membranes requires decreased CaMKII activity [131,132,133,134]. The reductions in CaMKIIα&β have been observed in the amyloid-beta precursor protein (APP)-overexpressing mice in an age-dependent manner [131]. Conversely, Reelin increases CaMKIIβ, which is critical for Reelin’s function of increasing spine density [137].

Reelin has been shown to affect APP processing and tau regulation. Reelin receptors interact with APP and APP-binding proteins and influence APP processing (reviewed [138,139]), and recent findings highlight a correlation between ApoER2 expression and neurofibrillary tangles development [107]. Of critical relevance to AD is the interaction of both APP and ApoER2 to Reelin [95], FE65 [140], Dab1 [141,142], and X11α/β [143]. FE65 and Dab1 have been shown to interact with APP and ApoER2/VLDLR through their NPxY domains. This interaction is facilitated by Reelin, which results in the increased cytoplasmic membrane retention of APP and the decreased amyloidogenic processing of APP [140,141,144]. The overexpression of Dab1 resulted in the same effect, with increased cell surface localization in the COS7 cells of APP and ApoER2, as well as increased α-secretase and decreased β-cleavage of APP [141]. Reelin’s interaction with APP has recently been shown to be necessary for increasing dendritic spine numbers in the hippocampus in vitro and in vivo [145]. Interestingly, the phosphorylation of FE65 (T579) by GSK3β can lead to its association with APP which, in turn, can facilitate Aβ production [146]. This, of course, is reduced with Reelin signaling, which both reduces GSK3β activity and increases APP’s association with ApoER2. Reelin’s central fragment, R3-6, has also been shown to directly interact with APP’s extracellular domain [95]. This association increased cell surface APP, increased APP α-cleavage, and reduced Aβ production [95]. Conversely, the binding of ApoE proteins to ApoER2 triggers the endocytosis of APP and, thus, increases Aβ production [143]. This is mediated by binding to X11α or -β, whose phosphotyrosine-binding domain interacts with APP and ApoER2. ApoE4 triggers the production of more Aβ than ApoE2 or 3 [143]. Reelin can interrupt the interaction between X11α/β and ApoER2, indicating another potential protective role of Reelin against Aβ toxicity [147].

Reelin and ApoER2 play an important role in directing dendritic complexity through the control of actin polymerization. Briefly, Reelin signaling via phospho-inositol 3 PI3 kinase induces phosphorylation of LIM kinase-1 (LIMK-1), which, in turn, phosphorylates cofilin at an inhibitory site, thus blocking the actin-depolymerizing activity of cofilin [148,149]. As a result, there is an increase in actin polymerization and dendritic spine growth with Reelin signaling, and mice that overexpress Reelin have a higher spine density and an increased spine complexity [150]. Conversely, Aβ has the opposite effect on actin microfilament dynamics. Aβ oligomers reduce the activity of LIMK and increase Slingshot activity, which, in turn, leads to the activation of cofilin, the depolymerization of actin filaments, reduced tau-mediated microtubule dynamics, and increased tau hyperphosphorylation and tauopathy [150,151,152]. In support of this, the genetic reduction of *cofilin* strongly mitigates tauopathy and synaptic plasticity deficits in Tau-P301S (PS19) mice [151].

### 2.4. Reelin in Schizophrenia

Schizophrenia (SCZ) is a devastating psychiatric disorder that affects approximately 1% of the population and is characterized by hallucinations, delusions, and, most importantly, cognitive disturbances. The first clinical features of SCZ typically emerge between early childhood and adolescence, with many patients experiencing chronic SCZ symptoms. Abnormalities in the Reelin gene (RELN) and its promoter, as well as the DAB-1 gene, have been identified in SCZ [153,154,155,156]. RELN gene polymorphisms (rs262355, rs362719, rs736707, rs7341475, Figure 1b) have been associated with SZ. Although some studies have contradictory results [157], this might be due to the number of individuals, population, or gender studied. Missense variants (c.9575 C>G) and deletions within the Reelin gene (C-terminal region exons 52–58) have all been associated with SCZ [157,158,159,160,161,162,163]. A recent genome-wide copy number variation analysis of Japanese schizophrenia patients identified a novel deletion in RELN-encoding Reelin [156,164]. Other genetic risk factors reported for SCZ include the proteins involved in Reelin signaling, APOER2 and DAB1. A genetic variant in the 3′ untranslated region (3′UTR) of APOER2 (rs5177) was associated with schizophrenia and other psychiatric disorders [165]. A single nucleotide variant (G382C) of the DAB1 gene has been hypothesized to increase susceptibility to SCZ [155].

Beyond these genetic changes, there is a consistent and reproducible reduction in Reelin protein and RNA in postmortem SZ brains [166,167,168], with a reduction of up to 50% in some regions of the brain [169]. In addition, decreased levels of Reelin, of its mRNA, or of Reelin’s regulatory transcription factor early growth response protein (ERG1) have been reported in the peripheral blood of schizophrenia patients when compared to healthy controls, and the levels were up-regulated following 12 weeks of treatment with antipsychotics [166,170,171]. Although peripheral blood levels of Reelin might not reflect the levels in the brain, these results are in favor of a dysregulation of the protein or its pathways in SCZ. The regulation of DNA’s methylation is an important factor that affects RELN expression, and it is now well established that patients with SCZ have higher blood levels of the RELN gene’s methylation compared to healthy controls, leading to a subsequent decrease in RELN expression in the methylated group [153,154,172]. Further investigation of the mechanisms of how methylation can regulate Reelin transcription and or/processing are needed and represent a therapeutic avenue to explore in the context of SZ.

Mouse models reflecting these genetic deletions result in animals that exhibit aspects of neuropsychiatric disorders [156,162,173,174,175]. Furthermore, dorsal forebrain-specific Dab1 knockout mice show symptoms such as hyperactivity and changes in their working memory, demonstrating that Reelin–Dab1 signaling is associated with SZ [176]. Mice carrying a deletion of exons 52 to 58 of RELN (*Reln*-del) showed several abnormalities in their cerebellar formation and in seeking behavior for social novelty, as well as impaired learning ability during reversal learning tasks [173,174]. In addition, the suppression or reduction of Reelin expression or of its downstream pathway in animal models exhibit features of SCZ, such as cognitive impairments, psychosis vulnerability, and learning deficits [64,177,178]. Conversely, the overexpression of Reelin protects against psychiatric disease-related phenotypes in mice [179]. Recent experiments using conditional gain and loss of function mouse models showed that the overexpression of Reelin leads to increased numbers of striatal interneurons and dopaminergic projections which could explain the positive outcome of increased Reelin levels on the SCZ phenotype [180].

A reduction in the number of Reelin-positive cells was also found in the hippocampus and cortex of a mouse model of prenatal-stress-induced SCZ. In this model, microinjections of recombinant Reelin protein into the hippocampus rescued the cognitive impairments while increasing the synaptic protein recruitment [181].

The HRM model is produced through the heterozygous Reelin mutation, resulting in a 50% reduction of the Reelin protein in the CNS throughout the development and the life of the animal. HRM juvenile mice show a reduced spinal density, an increased NMDAR sensitivity to Ro25-6981, a GluN2B-NMDAR antagonist, and reduced LTP [182]. The treatment of this model with Reelin recovered the biochemical, morphological, and physiological deficits in adult HRM [65]. Even more compelling was the recovery of HRM-related behavioral and cognitive impairments [65]. Recently, it was shown that the Reelin central fragment R3-6 could also recover behavioral deficits in HRM [56]. Neuromorphological, biochemical, and behavioral alterations occurring in HRM during development are persistent and could result in long-term effects on the adult phenotype. However, the Reelin supplementation data suggest that deficits associated with both HRM and possibly SZ may be due to tonic reductions in Reelin signaling in the adult and not exclusively a carry-over result of neurodevelopmental abnormalities.

### 2.5. Autism Spectrum Disorders

The Autism Sequencing Consortium identified RELN with a 95% probability of being a gene whose anomalies directly contribute to autism, and genome scans indicate a linkage of autism to the chromosome 7q21–q36 [183]. However, the use of RELN SNPs to determine susceptibility to ASD is still contradictory and may depend on the studied population. A meta-analysis for RELN rs362691 and rs736707 (Figure 1b) showed either a significant contribution to ASD [184] or no association [185], which could mean that the mutation in the RELN gene alone is not sufficient to induce ASD but can contribute to the pathogenesis of the disorder. Consistent with this hypothesis, 40 distinct, rare heterozygous variants have been identified in the RELN gene in ASD patients, mostly coming from neurotypical parents and not sufficient to cause ASD. Additional genetic or environmental events acting to reduce Reelin signaling might be necessary for the disorder to occur. Indeed, two recent studies identified three missense variants in the RELN gene (F10832-1, F11463-1 and F2688) that co-occurred with variants in genes in the Reelin cascade in ASD individuals [186,187]. A link between Reelin and autism also comes from recent studies indicating increased levels of Reelin in the plasma of children with autism [188]. In addition, decreased Reelin expression and signaling have been shown in the brain of adult patients with autism as well as in brain cells derived from these individuals. Some authors suggest that this discrepancy in Reelin levels between adults and children might be due to an age-dependent dysregulation of specific pathways and, in particular, on the epigenetic regulation of RELN through methylation of the RELN promoter. There is also a chance that Reelin signaling dysregulation occurs at an early stage in ASD and that the increased levels are due to a compensatory mechanism to a Reelin-resistant state, similar to what might be occurring with respect to Alzheimer’s disease.

Angelman syndrome (AS), an autism spectrum disorder, is a neuro-genetic disorder caused by a disruption of the imprinted and maternally expressed Ubiquitin-protein ligase E3A (UBE3A) gene that occurs approximately 1:12,000 live births [189,190]. Children with this disorder present with a developmental delay, a severe speech impairment, ataxia, a happy demeanor, and a high seizure propensity [191]. Currently, there is no approved treatment for AS. The AS mouse model was created using a null mutation of *Ube3a*, resulting in the loss of UBE3A expression, synaptic dysfunction, and disruption in both spatial and associative memory formation [192]. Specifically, there are severe hippocampal LTP deficits and impairments in memory formation associated with spatial learning via the hidden platform water maze and associative fear conditioning [192,193]. Although there is no consensus on the association of genetic variants of Reelin with AS (reviewed in [185]), the biochemical analysis of the CNS from both an AS human and an AS mouse show a reduction in the Reelin protein [69,194]. Similarly, the postmortem analysis of autistic subjects showed a reduction in Reelin and DAB1 mRNA in the cortex, together with an increase in VLDLR mRNA when compared to control subjects [69]. These data suggest that an impairment in Reelin signaling may be a contributing factor to AS and, possibly, a common feature in autism disorders. Similarly to the results seen with HRM, a single Reelin injection was able to recover the learning and memory defects and increase synaptic plasticity [194]. The increase in synaptic function achieved through Reelin supplementation is likely attributed to Reelin’s signaling effect on the post-synaptic neuron. As stated above, the Reelin signaling pathway is involved in the modulation of AMPAR insertion into the membrane, as well as the phosphorylation of NMDA receptors on the post-synaptic membrane that is most easily observed following short-term Reelin application [62]. In addition to receptor changes, Reelin increases spine density in wild-type apical dendritic spines [57]. A significant change in apical dendritic spine density was observed in the AS mice treated with Reelin [194]. It is possible that this modest increase in apical dendrites is sufficient to allow LTP recovery and may be coupled with alterations in receptor function.

### 2.6. Traumatic Brain Injury

Traumatic brain injury (TBI) is a major cause of death and disability. The primary cause of TBI is a direct mechanical force to the CNS, resulting in tissue damage, followed by a secondary injury due to a number of processes including blood–brain barrier breakdown and inflammatory responses from tissue damage. Two groups have recently shown reductions in Reelin levels after TBI. In a midline fluid percussion injury model in rats, Reelin decreased after TBI in the cortex and thalamus [195,196]. In a controlled cortical impact (CCI) model in mice, it was shown that Reelin expression decreased in the forebrain regions after TBI and that there was a decrease in the number of Reelin-expressing cells in the hippocampus [196]. Furthermore, these studies demonstrated in vitro that Reelin could protect hippocampal neuronal cells from glutamate-induced neurotoxicity. These data suggest that reductions of Reelin in TBI could cause hippocampal and cortical dysfunction, which ultimately contributes to cognitive impairments. Instead, increasing Reelin signaling may promote functional recovery and offer a novel therapeutic intervention for TBI.

## 3. Potential Therapeutic Interventions

We have presented how there is a strong association of Reelin in many neurodegenerative diseases, which suggests that Reelin may offer a potential therapeutic target for disease intervention. One potential area of therapeutic intervention could come from regulation of Reelin cleavage. As discussed above, published studies have discussed the fact that cleavage may regulate the function of the Reelin molecule [14,49,50]. Furthermore, the processing of Reelin may be altered with age and disease. Krstic et al. (2012) observed changes in protease fragments with age and changes in protease levels in the AD model 3xTg [7], suggestive of a dysregulation in Reelin processing. Studies of AD cerebrospinal fluid suggest a potential alteration in Reelin processing as well; however, the fragment analysis data are conflicting. One group observed an increase in C-terminal cleavage but no change in N-terminal cleavage [197], while others observed an increase in total Reelin and fragments indicative of increased N-terminal cleavage [100]. Some of these problems with analysis could depend on sample handling and preparation for analysis, due to Reelin’s instability and labile nature rending quantitative analysis difficult. Perhaps, they could also reflect changes in other post-translational modifications such as glycosylation. Further study is required to confirm changes in Reelin processing in disease and its regional cleavage within the brain. Targeting proteases with specific inhibitors has notoriously been difficult and often leads to unwanted off-target effects, which could reduce enthusiasm for this approach.

Since many diseases exhibit reduced Reelin levels, another area of potential intervention could be increasing Reelin expression and/or signaling. Targeting receptors for positive allosteric modulators or agonists may be one option but, currently, there are no established assays for Reelin downstream signaling, which would be amenable for high-throughput screening. An alternative approach could be to deliver the recombinant Reelin protein or to express it with gene therapy. Recombinant protein delivery would likely be expensive and difficult. Additionally, caution must be taken in increasing Reelin levels, since increasing Reelin at the periphery might have deleterious effects. In fact, anti-Reelin strategies are being studied for inflammatory diseases such as atherosclerosis and multiple sclerosis (reviewed in [32]). Therefore, a central approach would be more appropriate for neurological disorders. Gene therapy could be centrally delivered, but the large size of Reelin would restrict its use in adeno-associated virus vectors. The identification of smaller active fragments of Reelin protein could potentially offer an alternative solution. We recently published the identification of a combination of repeat regions 3 and 6 as one such potential candidate. This R36 fragment was identified from an ApoER2-luciferase complementation-binding assay [56] and was shown to be capable of Reelin signaling and of rescuing the HRM phenotype [198]; however, further work is needed to examine a gene-therapy approach.

## 4. Summary

Reelin signaling plays a critical role in neurodevelopment but also in adult synaptic plasticity. Indeed, there is increasing evidence of a role of Reelin in the adult brain on in learning and memory processes and that the disruption of Reelin signaling during aging and neurodegenerative diseases could contribute to cognitive impairments. Reelin signaling intervention in the brain may offer a therapeutic approach to a number of these diseases, with the advantage of Reelin signaling affecting numerous pathways controlling basal synaptic transmission and plasticity, spine morphology, and biochemical make-up and compartmentalization of major players in synaptic function. However, it is important to keep in mind that Reelin has a variety of peripheral effects and that increased peripheral levels of Reelin might affect the function of some organs and promote autoimmune diseases [32]. On the other hand, new therapies aimed at decreasing Reelin and Reelin signaling in inflammatory diseases should consider the effects of decreased Reelin signaling on the brain. Therefore, further investigation into the contribution of Reelin signaling during aging and in disease pathogenesis would certainly advance our understanding of related cognitive impairment and lead to new brain-specific therapeutic targets.

## Data Availability

The present manuscript is a review and therefore no new data were created.
